# New Vistas in the Biology of the Flagellum—*Leishmania* Parasites

**DOI:** 10.3390/pathogens11040447

**Published:** 2022-04-07

**Authors:** Scott M. Landfear

**Affiliations:** Department of Molecular Microbiology & Immunology, Oregon Health & Science University, 3181 SW Sam Jackson Park Road, Portland, OR 97210, USA; landfear@ohsu.edu

**Keywords:** flagellum, protein composition and function, flagellum attachment zone, flagellar membrane proteins, protein targeting to flagellum

## Abstract

Like other kinetoplastid protozoa, the flagellum in *Leishmania* parasites plays central roles throughout the life cycle. Discoveries over the past decade have begun to elucidate flagellar functions at the molecular level in both the insect vector stage promastigotes and intra-macrophage amastigotes. This focused review will highlight recent advances that contribute to understanding flagellar function in the various biological contexts encountered by *Leishmania* parasites.

## 1. Introduction

The flagellum is an organelle that has long been associated with motility in many eukaryotes, but more recently, it has been recognized to have both sensory [1] and secretory [2] functions. As their name suggests, hemoflagellate parasites, such as *Leishmania* and *Trypanosoma*, employ the flagellum for a wide variety of biological functions [3,4], including motility, attachment to host membranes in both the insect vector and mammalian host, interactions between parasites including mating and social motility, the secretion of vesicles, and sensation. This concise review will focus on a selected subset of discoveries related to flagellar structure and function in *Leishmania* spp. with particular emphasis on molecular characterization of this organelle, although relevant longstanding observations and some material from *Trypanosoma brucei* will be introduced where appropriate. The intention is to provide a short summary of recent advances at the cellular and molecular level and to suggest where new frontiers may emerge.

## 2. Structure of Flagellum throughout the *Leishmania* Life Cycle

**Figure 1:**
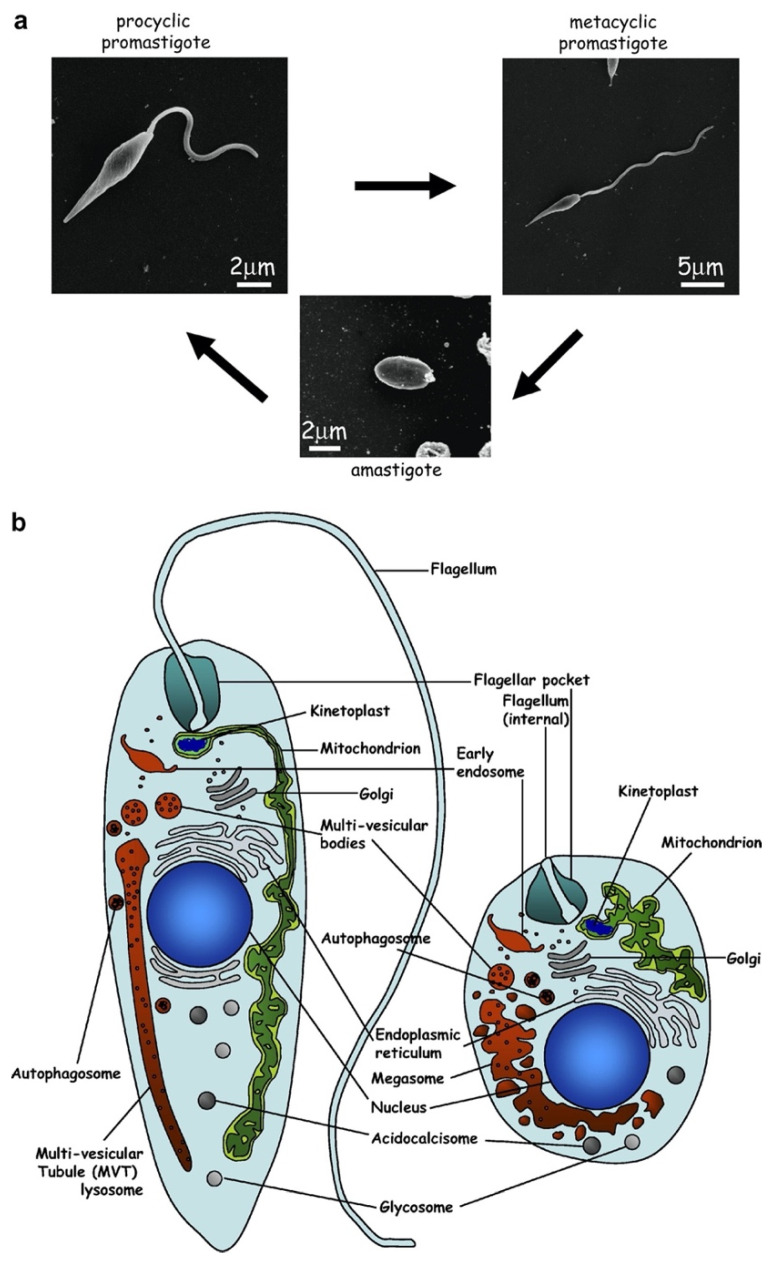
The flagellum throughout the life cycle of *Leishmania* parasites. (**a**) shows the flagellum from procyclic promastigotes, an elongated flagellum from infectious metacyclic promastigotes, and a short, barely emergent flagellum from amastigotes. (**b**) shows schematic images of a promastigote (left) and an amastigote (right) with the flagellum and flagellar pockets indicated. The figure was reproduced from reference [7] with permission.

One striking feature of the parasite life cycle is the extensive structural changes to the flagellum (Figure 1). Most notably, insect stage procyclic promastigotes have a long motile flagellum that emerges from the flagellar pocket at the anterior tip of the parasite. This appendage has typical features of a eukaryotic flagellum, including a basal region with 9 + 0 triplet microtubules and a basal body, followed distally by a transition zone, and then by the extended flagellar axoneme with standard 9 + 2 outer doublet and central pair microtubules. One unusual feature of the kinetoplastid flagellum is the presence of a paracrystalline array called the paraflagellar rod (PFR) that runs parallel to the axoneme from just outside the flagellar pocket to the distal tip, and this structure has been demonstrated, by deletion of the *PFR1* and *PFR2* genes encoding the most populous of the PFR proteins [5], to play an important role in parasite motility. As procyclic parasites develop into the mammalian infectious metacyclic forms within the sand fly vector, they acquire a more elongated flagellum and a greatly shortened cell body accompanied by increased motility. Most strikingly, when promastigotes invade mammalian host macrophages and transform into amastigotes, the flagellum shrinks in length so that it barely emerges from the tip of the flagellar pocket (FP). This gross morphological transformation is accompanied by the loss of the central pair of microtubules and the PFR, and the microtubules assume a 9 + 0 arrangement, with a disorganized pattern at the distal tip, designated 9 v, resembling the microtubule structure of a non-motile sensory cilium [6], and indeed, the amastigote flagellum is not motile. Strikingly, the short flagellum is often associated with the host cell parasitophorous vacuole membrane, and as such, it has been suggested that it may receive signals from the host and/or be involved in delivery of parasite components to the infected macrophage.

For a significantly more detailed structural study of the promastigote and amastigote flagellum and flagellar pocket, readers are referred to the comprehensive serial section electron tomography study by Gull and colleagues [8]. This work revealed the three-dimensional structures of these organelles and the arrangement of many suborganellar components.

## 3. Comprehensive Proteome of the *Leishmania* Flagellum

A recent major advance in dissecting the *Leishmania* flagellum is the comprehensive proteomic analysis carried out by Gluenz and colleagues [9]. These authors first developed an improved method for isolation of the flagellum from *L. mexicana* promastigotes employing a calcium shock followed by mechanical shearing and density gradient isolation of highly enriched flagellar (separated at the point of exit from the cell body) and deflagellated cell body fraction. These fractions were further resolved into detergent-soluble and -insoluble components, and the fractions were subjected to quantitative label-free proteomic analysis to identify proteins enriched in each fraction. This analysis identified hundreds of probable flagellar proteins, both cytoskeletal and some integral membrane polypeptides, and many previously known flagellar components were among these lists, validating the results. An interactive website (www.leishgedit.net/leishgedit_db, accessed on 6 April 2022) has been developed by these authors that reports proteins selectively enriched in both the detergent-soluble and non-soluble fractions of the flagellum versus the cell body, and readers can access this site for a comprehensive identification of flagellar proteins.

The authors proceeded to knock out about 100 genes encoding flagellar proteins employing the high-efficiency CRISPR/Cas9-mediated technology they had pioneered previously [10]. This knockout library allowed the rapid assessment of biological functions for both previously known and novel flagellar proteins. A major phenotypic metric was motility of knockout promastigotes, resolved into both speed and directionality, and the mutant lines were clustered into five groups: unaltered motility, fast, slow, uncoordinated, and paralyzed. All motility mutants were viable in vitro, including those that failed to assemble a flagellum, a characteristic of several mutants in intraflagellar transport (IFT) machinery [11] that mediates transport of proteins along the flagellum in both anterograde and retrograde directions. Of particular interest, these mutants allowed a test of the previously unproven assumption that promastigote motility is required for development in the sand fly, where migration between distinct anatomical locations occurs in a reasonably choreographed pattern [4]. By infecting *Lutzomyia longipalpis* sand flies with both mixed populations of bar-coded null mutants and discrete infections with specific mutants, the results demonstrated that paralyzed mutants are strongly impaired in development within the insect, with most parasites excreted during defecation and the few remaining ones restricted to the midgut. Uncoordinated swimmers were also largely lost by day 6 of infection, with the remaining parasites also restricted to the fly midgut and unable to migrate forward to the mouthparts and stomodeal valve. This detailed analysis strongly suggests that the directional motility of promastigotes is essential for development within the insect vector and likely required for successful transmission from the fly to the vertebrate host.

More recently, Beneke et al. [12] also used the isolation of detergent-extracted flagellar axonemes, proteomics, and CRISPR/Ca9-mediated gene knockouts to study the role of a tripartite component of the tether and tether head (T/TH) complex, key regulators of dynein function in the flagellum. Knockouts in individual components, including the novel LAX28 protein, were reduced but not completely defective in speed and directionality of movement and had somewhat curled flagella.

Overall, the above corpus of work illustrates the power of (i) identifying the complex cohorts of proteins within an organelle and (ii) applying high throughput genetic methods, such as gene knockout libraries, to elucidate the structure and function of an organelle and of individual protein components. The work summarized here and in several other sections of this review was conducted in *L. mexicana*. Although most results are likely to apply widely across diverse *Leishmania* species, there are biological and genetic differences between species, and some significant distinctions in the function of flagellar proteins may emerge as these organellar components are explored more broadly.

## 4. Identification of the Flagellum Attachment Zone

The flagellum attachment zone (FAZ) is a prominent morphological feature of *T. brucei*, where it represents an adhesion between one face of the flagellum and the cell body membrane that extends almost the entire length of the flagellum [13]. This structure consists of many components, including those in the lumen of the flagellum (e.g., ClpGM6), in the flagellar membrane (FLA1BP), in the cell body membrane (FAZ5, FLA1), and in the cytosol of the cell body (FAZ1). A principal purpose of the FAZ in trypanosomes is to position the flagellum at its lateral site of attachment to the cell body and to act as a ‘cellular ruler’ regulating cell length and organelle position. It is essential for cell division and for differentiation between life cycle stages.

In contrast, *Leishmania* parasites exhibit a flagellum that is free from the cell body (Figure 1) and thus were thought for years not to contain a FAZ. Remarkably, once molecular components of the *T. brucei* FAZ were identified, many orthologous proteins were discovered within the genomes of *Leishmania* parasites [8]. The tagging and localization of these proteins in *L. mexicana* promastigotes showed that they all localized to a more-or-less discrete spot at the base of the flagellum, albeit with somewhat distinct positioning for different components. High-resolution localization indicated that the same four domains of the FAZ (flagellar lumen, flagellar membrane, cell body membrane, cell body cytosol) existed in *L. mexicana*, but the structure was restricted to a discrete adhesion between the base of the flagellar membrane and a site toward the anterior of the flagellar pocket membrane. Thus, the FAZ is radically different between the two parasites, raising the issues of what functions it serves in *Leishmania* and how it is constructed so differently despite sharing many orthologous proteins.

**Figure 2:**
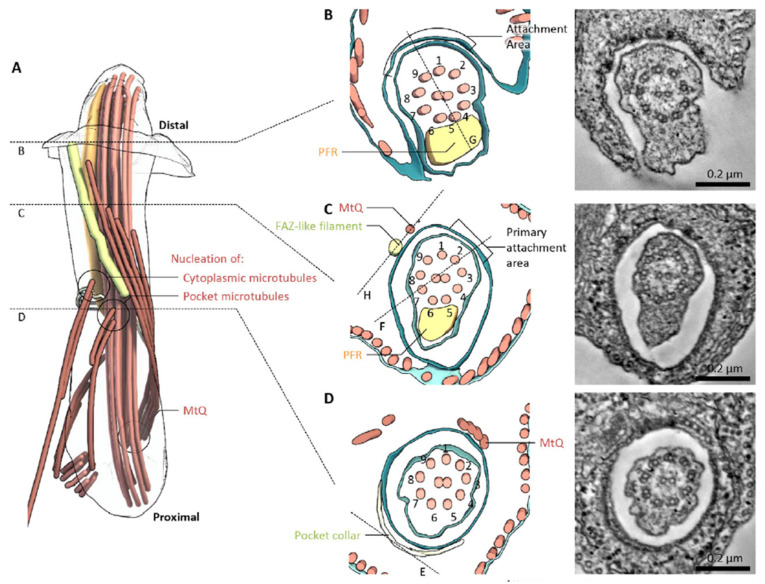
Model of the *L. mexicana* promastigote FAZ based upon serial section electron tomography. (**A**) Overview of non-membrane structures in the flagellar pocket. (**B**–**D**) Cross sections of the flagellum and flagellar pocket at various planes indicated in (**A**). This figure was reproduced from reference [8] with permission.

Serial section electron tomography was applied to the FP region of both promastigotes and amastigotes to develop a three-dimensional model of this region, including the FAZ (Figure 2). The model proposes that the FAZ cytosolic elements assemble into a rod (yellow in Figure 2A) that runs parallel to the FP membrane and terminates near the anterior opening of the FP. This rod may contain FAZ1, FAZ2, FAZ5, and FAZ8, although the association of these proteins with the rod has not been demonstrated directly. The flagellar membrane and FP membrane adhere, but the adhesion does not closely correspond to the location of the FAZ filament. Adhesion is likely mediated by FAZ proteins that project from either membrane into the intermembrane space, and the FM localized FLA1BP is proposed to be associated with the regions of intermembrane attachment. Sections perpendicular to the FP membrane showed that these areas of close membrane attachment contained more-or-less regularly spaced electron dense junctional complexes that mediate membrane–membrane attachment (Figure 2B,C). In amastigotes, the pocket and FAZ showed some distinct features. For instance, the regions of close FP and FM apposition did not exhibit discrete junctional complexes but rather had extended areas of electron dense membrane adhesion and a clear constriction in the FM. As with promastigotes, amastigotes showed a FAZ filament on the cytosolic side of the FP membrane. Wheeler et al. suggest that the *L. mexicana* FAZ may be involved in determining the proper propagation of cell structure and relative organelle localization during cell division, as it is in *T. brucei* [13].

Understanding of FAZ function was further probed by the deletion of the gene encoding FAZ5, a polytopic protein integrated into the FP membrane component of the FAZ [14]. The deletion of this single FAZ protein alters the FP structure in amastigotes so that the pocket becomes more bulbous, and there is a shorter region of close apposition between the FP and FM membranes in the neck region of the FP. Furthermore, the electron-dense junctions between these two membranes in the neck regions clearly seen in wild type promastigotes are replaced by a limited number of electron-dense regions that are no longer associated with attachment regions. Although promastigotes in culture do not have diminished viability, Δ*faz5* null mutants were able to initially infect sandflies and populate the endoperitrophic space within the midgut on days 1–2, but by days 6–8 post-infection, few sand flies were still infected, and among those that were, the parasites did not migrate forward to the stomodeal valve but remained in the endoperitrophic space and anterior midgut. Hence, the development of the parasite within the sand fly is severely compromised and presumably would prevent transmission from the fly to an animal. Infections of mice with the Δ*faz5* null mutant led to very small footpad lesions, compared to wild-type or add-back lines, and to greatly reduced numbers of parasites within the footpad tissue or draining lymph nodes, establishing that virulence is strongly impaired in the absence of FAZ5. The reasons for poor survival of Δ*faz5* amastigotes in animals are not certain, but the authors suggested it could have to do with the virtual disappearance of the tightly apposed FP neck region in null mutant amastigotes. This morphological alteration may leave amastigotes in the animal vulnerable to import harmful material from the host into the FP, resulting in the impaired long-term viability of amastigotes. Overall, these studies confirm the critical role of the FAZ in both promastigotes and amastigotes in vivo and underscore the essentiality of this structure throughout the parasite life cycle.

Subsequent analysis of a Δ*faz2* null mutant [15] uncovered a distinct and intriguing phenotype. These mutants had a disrupted membrane organization at the anterior cell tip in promastigotes that caused daughter cells to remain connected to each other by a membranous connection between each flagellum. Serial electron tomography of the null mutant revealed a much shorter FAZ filament and greatly reduced FM attachment to the FP membrane. The FAZ2 null mutant was also unable to proliferate and develop within the sand fly, and it was greatly reduced in virulence in the mouse. Thus, although different FAZ proteins have some important distinctions in phenotypes when they are deleted, the critical role of the FAZ has now been established using different molecular components.

Another component of the FAZ, FAZ7B, was also found to play an important role [16]. This is the only FAZ protein that contains a kinesin motor domain, and it is localized on the cytosolic domain of the FAZ. The deletion of the *FAZ7B* gene impaired the growth of both promastigotes and amastigotes in vitro and caused altered cell division, flagellar length, and FP structure and function, and an intact conserved kinesin domain was required for wild type phenotypes to be preserved. In particular, the arrangement of components of the FP collar, a ring-like structure surrounding the FP membrane on its cytosolic side, was altered in the knockout. The FAZ7B null mutant was also strongly impaired for replication and migration in the sand fly and for virulence in mice, again confirming the importance of the FAZ in vivo in both insect and vertebrate hosts.

## 5. Targeting and Functions of Membrane Proteins in the Flagellum

Most work on the *Leishmania* flagellum has focused on the cytoskeletal components, with impressive advances in recent years. Less is understood about the molecular components of the FM, both about how they are targeted to this organelle and what functions they play during the parasite life cycle. Nonetheless, it is clear that the flagellar membrane plays a variety of vital roles for the biology of the parasite [3] and likely mediates critical interactions between the parasite and both its insect vector and vertebrate hosts. Hence, studies on FM proteins constitute an important frontier in *Leishmania* research.

One striking example of differential transport between the flagellar and cell body membranes emerged from studies in our laboratory on three glucose transporter isoforms of *L. mexicana.* Whereas the GT2 and GT3 isoforms trafficked to the cell body plasma membrane and were excluded from the FM, the GT1 isoform was localized largely in the FP and FM [17]. Null mutants in the *GT1* gene experienced a catastrophic drop in viability as promastigotes grew to high density and depleted glucose from the medium, suggesting that flagellar GT1 may serve a role as a glucose sensor required for successful transition of parasites from logarithmic to stationary phase [18]. Deletion and site-directed mutagenesis identified a short sequence within the unique N-terminal hydrophilic domain of GT1 that was required for FM targeting [17], and subsequent TAP tagging identified a kinetoplastid-specific protein, KHARON, that interacted with this signal and was required for efficient FM targeting of GT1 [19]. Although KHARON is found in abundance in the subpellicular cytoskeleton surrounding the cell body and in the mitotic spindle, it also localizes at the base of the flagellum cytoskeleton, where it most likely acts to mediate the passage of GT1 from the FP membrane into the FM, and Δ*kharon* null mutants fail to traffic GT1 to the FM and accumulate this protein in the FP membrane. A KHARON ortholog is also expressed in *T. brucei*, where it is required for FM targeting of a calcium channel [20]. Recently, high-resolution mapping of *T. brucei* KHARON on the flagellar cytoskeleton (M. Sanchez and S.M. Landfear, unpublished results), using deconvolution microscopy and marker proteins with known positions, established that KHARON is located at the interface between the basal body (BB) and the transition zone (TZ). The TZ is a complex structure just distal to the BB [21] that mediates the entry of proteins, likely both luminal and membrane, into the flagellum. Hence, the recent data suggest that KHARON may usher FM cargo into the TZ by physically interacting with that cargo and bringing it to a position between the transition fibers (TFs) [22] at the distal end of the BB and the proximal frontier of the TZ. Nonetheless, the binding of cargo such as GT1 to KHARON must be reversible, as KHARON does not appear to traffic along the axoneme but stays at the BB/TZ interface, whereas GT1 is distributed along the length of the FM. The observation that intraflagellar transport (IFT) particles are recruited and concentrated immediately distal to the TFs [23,24,25], more or less coincident with KHARON, raises the possibility that KHARON could first capture FM cargo and then transfer it to these adjacent multi-subunit complexes for transport past the TZ, but this model remains to be tested. In mammalian cells, IFT-A particles mediate the entry of some mammalian G-protein coupled receptors into the primary cilium [26], so KHARON could guide GT1 into an analogous trafficking pathway.

Parallel studies on other FM proteins have established that different FM trafficking machinery must exist that is distinct from KHARON. Thus, localization of FLA1BP to the FM component of the FAZ or of a membrane bound cAMP-phosphodiesterase [9] and an aquaporin, AQP1 [27], along the FM was not disrupted in Δ*kharon* null mutants [28]. Similarly to the identification of KHARON as an interacting protein required for FM trafficking of GT1, the identification of proteins that interact with these other FM proteins and are required for their correct trafficking is likely to identify other FM trafficking machinery.

A variety of other FM proteins have also been identified. The flagellar aquaporin, AQP1, was discovered in *L. major* [27] and shown to mediate flux of water, osmolytes, and arsenates and promote the uptake of and sensitivity to anti-parasitic arsenicals [29,30]. The overexpression of AQP1 increased osmotaxis of promastigotes toward high concentrations of glucose and improved volume recovery following osmotic shock in both promastigotes and amastigotes [27]. These results suggest that this channel could play an important role in sensory adaptation of the parasite to varying osmolar conditions experienced in the insect and macrophage. Additionally, it may carry out an important sensory function by promoting migration of promastigotes from the midgut to the proboscis of the sand fly, a process that is thought to be dependent upon osmotaxis [31], but this role has not yet been tested experimentally. An arginine transporter from *L. donovani*, LdAAP3, was shown to localize to both the FM and glycosomes [32]. Its expression level was upregulated by arginine starvation, indicating that the parasite senses extracellular levels of this amino acid, and the response to arginine starvation was dependent upon a MAP kinase-2 signaling cascade. The upregulation of LdAAP3 is part of a broader arginine deprivation response that regulates the expression of a cohort of proteins, but the arginine sensor and the binding site on the transporter are distinct [33].

SMP-1 is a small palmitoylated/myristoylated protein that attaches to the luminal face of the FM via its lipid anchor [34], and dual palmitoylated/myristoylated proteins such as the flagellar calcium binding proteins, FCaBPs, have been shown to traffic to the FM in trypanosomes [35], where they may respond to calcium transients. It has been proposed that such lipid-anchored proteins may partition into the FM by association with lipid raft-like components of the FM, an organellar membrane that is enriched in sterols, sphingolipids, and glycolipids compared to the cell body plasma membrane [36]. However, additional studies [35] have demonstrated that, although the first 12 amino acids of the *T. cruzi* FCaBP are sufficient for dual lipid anchor addition, the first 24 amino acids are required for FM targeting, thus indicating that lipid anchor addition is not the sole determinant of flagellar targeting. Other integral FM proteins include various adenylate cyclases in both *Leishmania* promastigotes [9] and *T. brucei* bloodstream [37] and procyclic forms [38]. A number of distinct patterns of localization were observed for some of these proteins along the length of the FM in trypanosomes, being determined by sequences near the C-terminus. In general, specific signaling pathways for these proteins have not yet been established, but a role has been demonstrated for trypanosome bloodstream form adenylate cyclases in countering the host innate immune response [39] and for procyclic adenylate cyclases in the process of social motility [40]. In particular, what activates or inhibits the synthesis of cAMP by these adenylate cyclases is poorly understood. Nonetheless, they are likely involved in sensory signaling and are an intriguing family of proteins for future studies both regarding trafficking and function.

## 6. Summary and Perspectives

The past decade has witnessed pronounced advances in our understanding of the *Leishmania* flagellum, an organelle with a wide range of functions during the parasite life cycle. Particularly promising is the application of high-throughput approaches, such as quantitative proteomics and focused gene knockout libraries, to identity and elucidate the functions of an array of flagellar proteins. Unexpected discoveries, such as the existence and functional importance of the FAZ, have also infused novelty into this discipline. Many vistas remain to be explored. One of the most interesting and challenging frontiers involves the roles of the short amastigote flagellum, particularly its potential activities in sensing the environment inside the macrophage and delivering parasite effectors to the host. Another is to explore specific sensory functions of *Leishmania* flagella. A recent report [41] demonstrated that infectious metacyclic promastigotes switch from ‘run and tumble’ motility to directed motion once they encounter macrophages, suggesting host-directed chemotaxis. The existence of flagellar adenylate cyclases and phosphodiesterases suggests a likely role for cAMP signaling in sensation, but what the initial stimuli or ultimate readouts of these pathways might be remains obscure. The presence of transporters and channels in flagellar membranes raises the possibility that they are involved in sensing the changing environment, but solid evidence remains scarce. The nature of molecular interactions between the flagellum and sand fly tissues or macrophage membranes is still largely undefined. Some advances have been made regarding trafficking or tethering mechanisms for luminal or membrane components of the flagellum, but our understanding is still in its early phase. The continually improving molecular, genetic, and microscopy tools that can be applied to these parasites suggest that the near future will be replete with many important and novel discoveries regarding this multi-functional organelle.

## Data Availability

Not applicable.

## Abbreviations

BB: basal body; IFT: intraflagellar transport; FAZ: flagellum attachment zone; FM: flagellar membrane; FP: flagellar pocket; PFR: paraflagellar rod; TF: transition fiber; TZ: transition zone.
